# Reply: Cardiac Biomarkers in Patients With Suspected Amyloid (Transthyretin) Cardiomyopathy: Which Cut-Offs?

**DOI:** 10.1016/j.jaccao.2025.04.001

**Published:** 2025-06-17

**Authors:** Laura De Michieli, Omar F. AbouEzzeddine, Angela Dispenzieri, Martha Grogan, Allan S. Jaffe

We appreciate the comments from Dr Aimo and colleagues regarding our study using high-sensitivity cardiac troponin T and N-terminal pro–B-type natriuretic peptide in patients undergoing ^99m^Tc pyrophosphate (PYP) scintigraphy for possible transthyretin amyloid cardiomyopathy.^1^ We welcome the opportunity to compare our findings.^2^ We explored lower thresholds to avoid false-negative results, but we did not find thresholds to rule in the disease. The combination of high-sensitivity cardiac troponin T <14 ng/L and N-terminal pro–B-type natriuretic peptide <180 ng/L provided similar performance, with some false negatives. In our study, the prevalence of transthyretin amyloid cardiomyopathy was 30%^1^; in contrast, that of cardiac amyloidosis was 60% in the study reported by Vergaro et al.^2^ Any set of metrics to optimize sensitivity and specificity will provide different predictive values depending on the cutoffs chosen and the pretest probability. When the pretest probability is lower, it is important for clinicians to consider that metrics will provide different answers from those obtained in centers with a high prevalence of disease.

Heterogeneity in patients’ characteristics and diagnostic work-up (including different tracers) may have a role in the differences observed. Regarding imaging, ^99m^Tc PYP scintigraphic adjudication was performed per the Mayo Clinic’s internally validated protocols, which are similar in performance to Perugini grading and as previously reported.^3^ This methodological difference is unlikely to have much influenced the findings. Differences in diagnostic algorithms and studies design are relevant. Our cohort consisted of patients undergoing ^99m^Tc PYP scintigraphy for clinical practice, and all were evaluated for monoclonal component.^1^ As per the U.S. expert consensus document,^4^ the work-up for light-chain (AL) amyloidosis does not consistently include ^99m^Tc PYP scintigraphy, hence the lower prevalence of AL amyloidosis in our population. However, some of our patients were referred from different institutions, and all the testing might be performed during the same day. Thus, 15 patients with positive results on ^99m^Tc PYP scintigraphy did have AL amyloidosis, as did about 6% of the entire cohort (with negative results on ^99m^Tc PYP scintigraphy). This is lower than reported by Vergaro et al^2^ and might have influenced the results.

Further studies using a standardized approach to patient selection and diagnostic evaluation would be ideal to validate diagnostic cutoffs. However, even with uniform processes of care, the prevalence of the disease will still be problematic. Clinicians will need to interpret their local results with this understanding in mind.
